# Supplementary material for: Pain burden, sensory profile and inflammatory cytokines of dogs with naturally-occurring neuropathic pain treated with gabapentin alone or with meloxicam
Source: PLoS One. 2020 Nov 30;15(11):e0237121. doi: 10.1371/journal.pone.0237121 (PMC7703878; doi:10.1371/journal.pone.0237121)
Supplement: S1 File — (DOCX) [file pone.0237121.s001.docx]

**Gabapentin in dog serum**

1. ***Analytical Procedure***
   1. *Reagents*

Gabapentin and ^2^H_6-_gabapentin were purchased from Toronto Research Chemical (Toronto, ON, Canada). Drug-free dog serum was supplied by our laboratory. Formic acid was purchased form Sigma-Aldrich (St-Louis, MO, USA). Other chemicals, including, methanol, acetonitrile and water were purchased from Fisher Scientific (Fair Lawn, NJ, USA).

- 1. *Sample preparation*

Using protein precipitation as sample preparation technique, gabapentin was extracted from dog serum. One thousand microliters of internal standard solution (100 ng/mL ^2^H_6-_gabapentin in methanol) was added to an aliquot of twenty-five microliters of sample. The sample was vortexed for approximately 5 seconds and let stand for a period of 10 minutes, then centrifuged at 16 000 × *g* for 10 minutes. The supernatant was transferred into a clean 13 x 100 mm borosilicate tube and evaporated to dryness at 40⁰C under a gentle stream of nitrogen. The dried extract was re-suspended with 2000 µL of 0.1% (v/v) formic acid in water and transferred to an injection vial for analysis.

- 1. *Chromatographic conditions*

A gradient mobile phase was used with a Thermo Scientific Aquasil C18 analytical column (100 x 2.1 mm I.D., 5 µm) operating at ambient temperature. The initial mobile phase conditions consisted of 0.1 % (v/v) formic acid in acetonitrile and 0.1 % (v/v) formic acid water at a ratio of 5:95, respectively, and this ratio was maintained for 0.5 min. At 0.6 min, a step gradient was applied to a ratio of 95:5 and maintained for 2.9 min. At 3.6 min, the mobile phase composition was reverted to the original conditions and the column was allowed to equilibrate for 2.4 min for a total run time of 7.0 min. The flow rate was fixed at 200 µl/min and both compounds eluted at 3.1 min.

- 1. *Mass spectrometric conditions*

The mass spectrometer was interfaced with the UHPLC system using a pneumatic assisted heated electrospray ion source. MS detection was performed in positive ion mode, using selected reaction monitoring (SRM). In order to optimize the MS/MS parameters, standard solutions of gabapentin and ^2^H_6_-gabapentin were infused into the mass spectrometer. The following parameters were obtained. Nitrogen was used for the sheath and auxiliary gases and was set at 50 and 15 arbitrary units. The HESI electrode was set to 3500 V. The capillary temperature was set to 350⁰C and the vaporizer temperature was set to 400⁰C. Argon was used as collision gas at a pressure of 2.5 mTorr. The precursor-ion reaction for gabapentin and ^2^H_6_-gabapentin were set at 172.2 🡪 137.3 and 178.3 🡪 143.2, respectively. The collision energy (E_lab_) for both compounds was set to 15 eV. Total cycle time was set at 0.25 seconds. Peak width of Q1 and Q3 were both set at 0.7 FWHM.

1. ***Chromatograms***

The mass chromatograms of the extracted blank serum sample did not show any significant interference from endogenous substances at the expected retention time of gabapentin or ^2^H_6_-gabapentin.

1. ***Analytical Qualification***

A stock solution of gabapentin was prepared by accurately weighing and dissolving the compound in water to obtain a final concentration of 0.5 mg/mL. A serie of standard working solution of gabapentin was obtained by mixing the standard stock solution and further diluting with water. Calibration standards were prepared by fortifying the dog serum with the standard working solutions at 5% (v/v) to enable concentrations spanning the following analytical range 0.10 to 25.0 µg/mL. The method is linear using a linear regression weighted 1/x analysis. R^2^ ≥ 0.9988 for the qualification batch.

1. ***Sample Analysis***

During qualification, the method met all requirements of sensitivity, linearity, precision and accuracy within a batch. This assay is suitable for the analysis of gabapentin in dog serum. The correlation coefficient for gabapentin during all sample analysis batches was greater than R^2^ ≥ 0.9997. During all analytical batches, the accuracy ranged from 100.1 to 106.7 % and the precision observed was greater than 1.4 %. Samples were injected in duplicate. Dogs from the study with detectable concentrations of gabapentin at initial presentation and during the placebo period were repeated, the repeat results confirmed initial analysis.
